# Access to Medical Residency: A Qualitative Study of Medical Graduates' Experiences in Georgia

**DOI:** 10.1177/23821205251342050

**Published:** 2025-05-11

**Authors:** Mariam Kirvalidze, Mariam Kasrashvili, Aleksandre Tskitishvili, Giorgi Aladashvili, Nikoloz Chelidze, Nikoloz Tvildiani, Karsten Lunze, Ilia Nadareishvili

**Affiliations:** 1 Aging Research Center, Department of Neurobiology, Care Sciences and Society, Karolinska Institutet and Stockholm University, Stockholm, Sweden; 2 256085 David Tvildiani Medical University, Tbilisi, Georgia; 3 12259 Boston University, Chobanian and Avedisian School of Medicine, Boston, MA, USA; 4 1836 Boston Medical Center, Section of General Internal Medicine, Department of Medicine, Boston, MA, USA

**Keywords:** residency, medical training, enrollment, Eastern Europe

## Abstract

**Objectives:**

Strategic planning for the health workforce–particularly in emerging middle-income countries like Georgia–is essential for maintaining an effective healthcare system. Medical residency training is crucial for developing a well-rounded healthcare workforce equipped with the competencies needed to deliver high-quality care and maintain a balance of specialties. Understanding the enrollment process and experiences of medical graduates in residency programs can help identify areas for improvement. These insights can inform interventions to develop a physician workforce that aligns with population needs and remains responsive to the evolving healthcare system. We aimed to explore the experiences of medical graduates navigating Georgia's residency enrollment process, as well as residents’ postgraduate training experiences, to identify key areas for improvement. Additionally, we examined attitudes towards enrolling in residency programs abroad to better understand the potential impact on the ongoing phenomenon of “brain drain.”

**Methods:**

This study employed a qualitative research design based on individual interviews, conducted via Zoom. We explored the experiences and perceptions of residency enrollment among a purposefully selected sample of 10 participants using reflexive thematic analysis.

**Results:**

We identified six overarching themes. Participants described the placement exam as being of suboptimal quality and reported challenges in navigating the enrollment process, often accompanied by emotional stress. Working in residency without a salary was considered unfair and posed an economic burden. The absence of structured mentorship further contributed to dissatisfaction, prompting several participants to consider alternative career paths or pursue residency opportunities abroad.

**Conclusion:**

Georgia's medical residency enrollment process requires greater transparency and reduced burdens on aspiring professionals to attract the most qualified candidates. Policy reforms and strategic initiatives should promote socioeconomically equitable access to residency programs and address concerns related to “brain drain,” ultimately supporting the development of a sustainable healthcare workforce.

## Introduction

Georgia, a post-Soviet, upper-middle-income country with an estimated population of 3.7 million, faces numerous challenges in its efforts to improve population health through multisectoral development, health system strengthening, and the pursuit of universal health coverage. Effective planning and management of human resources for health (HRH) are essential to achieving these objectives efficiently. However, frequent changes in government-appointed officials and the absence of long-term strategic planning have contributed to the suboptimal mobilization of the health workforce.^1^ Medical residency training represents a critical step in producing the right number of well-trained physicians with the competencies needed to meet population health needs across various specialties. In Georgia, the regulation and oversight of medical residency training and related processes fall under the responsibility of the Ministry of Internally Displaced Persons from the Occupied Territories, Health, Labour, and Social Affairs of Georgia (MoH).

Undergraduate medical education in Georgia is offered through a large number of state and private medical universities and programs. Several of these programs provide instruction exclusively in English, primarily targeting international students. Earning an undergraduate MD degree requires the completion of at least 360 *European Credit Transfer and Accumulation System (ECTS)* credits and typically spans six years. To pursue postgraduate training in a medical residency, MD graduates must first pass the *Unified Postgraduate Qualification Examination*^2,3^ Upon passing the exam, the applicants must then enroll in a state or private medical facility that is accredited to offer residency training.

**Figure 1.**
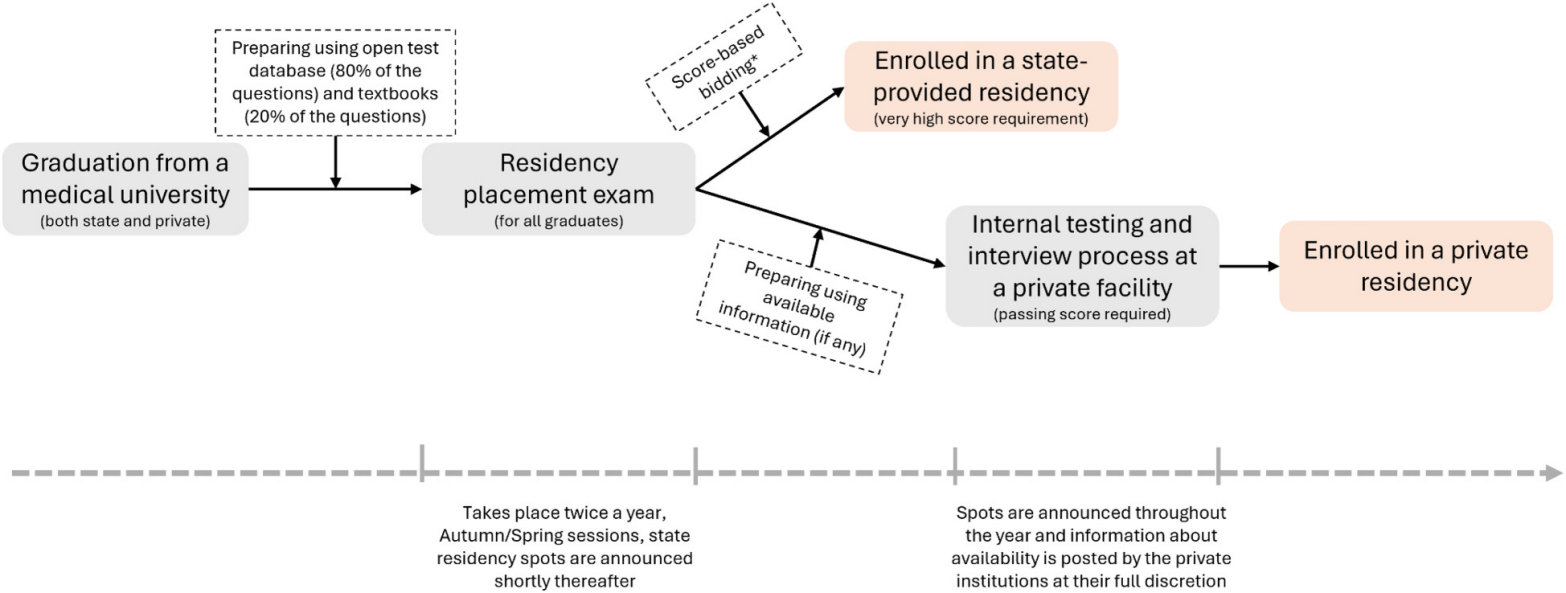
Flowchart Describing the Process After Graduation to Residency Program Enrollment.

To provide the country-specific educational context for Georgia, Figure 1 outlines the medical residency enrollment process. The *Unified Postgraduate Qualification Examination* contains 200 multiple-choice questions drawn from two sources: an “open” question bank–where questions and correct answers are publicly available for “preparation” and account for 75%–80% of the exam–and a “closed” set of questions, which make up the remaining 20–25% and vary between exam sessions. Following the exam, the enrollment process differs depending on whether a candidate is applying to a state or private residency program. For state programs, candidates use an electronic platform to “bid” for available positions based on their exam scores. This system displays all bids, promoting transparency. In contrast, private programs require applicants apply to specific calls for applications issued by accredited facilities and often involve additional internal examinations and interviews conducted by admission officials. Private programs generally have lower *Unified Postgraduate Qualification Examination* score requirements—sometimes accepting any passing score—and enjoy greater discretion in announcing and filling residency positions, within broad guidelines set by the MoH. Both state and private residency programs are tuition fee-based, and residents do not receive a salary. Tuition fees vary significantly, with private programs fees generally higher than those state-funded programs.

*In the event of identical scores, an interview is scheduled for the tied applicants. In addition, if the applicants have passed United States Medical License Examination steps 1,2 and 3, they are automatically granted 200 points (the maximum) and can compete with others with this score.

A report by the *State Audit Office of Georgia* assessing the country's health workforce,^4^ identified several major challenges related to the quality of medical residency education. According to the report, 65% of facilities with accredited residency programs failed to submit annual reports required for quality assurance; half of the active residents interviewed by auditors expressed dissatisfaction with the quality of the training; 69% of interviewed residents reported having to seek external employment to cover the cost of residency tuition; and a substantial number of residency examinations had not been updated for over a decade.^4^ Given the findings of this report, the importance of residency training for health workforce planning, widespread concerns about fair and equitable access to medical residency in Georgia reported in the media,^5,6^ and the lack of published studies on the topic, we aimed to conduct the first qualitative study exploring the experiences of residency-seekers and current residents regarding the residency entry exam and enrollment process in Georgia. The objectives of this study were to: 1) explore the experiences of medical graduates in navigating the residency enrollment process; 2) understand the emotional, psychological, and practical aspects involved in preparing for and participating in residency placement examinations and enrollment; 3) identify areas for improvement within the residency enrollment process; and 4) explore attitudes towards opportunities to enroll in residency programs abroad (ie, “brain drain”).

## Materials and Methods

### Study Design

This qualitative study was based on individual interviews conducted in Georgia during the spring of 2024. Data were analyzed using reflexive thematic analysis^7^ to explore the experiences and perceptions related to the residency enrollment process among a sample of 10 participants. Ethical approval for the study was obtained from the David Tvildiani Medical University Ethics Committee (IRB00014578, Dnr#1/24.) The reporting of this study adheres to the Consolidated Criteria for Reporting Qualitative Research (COREQ),^8^ and the completed checklist is provided as Supplemental Table 1.

### Participants

Participants were recruited via Facebook support groups dedicated to medical residency training in Georgia. These groups are widely used by medical graduates seeking residency placements and serve as key platforms for sharing information among peers. Participants were purposefully selected based on their familiarity with the enrollment processes and willingness to share their experiences. Efforts were made to ensure adequate representation in terms of gender, place of residence (within and outside the capital, Tbilisi), year of graduation, and type of undergraduate university or residency program (private vs state). The inclusion criteria were: 1) having taken the *Unified Postgraduate Qualification Examination* at least once, and 2) either currently being enrolled in a residency program or attempting to secure a residency placement in Georgia.

### Data Collection

Individual interviews were conducted with each participant via Zoom (Zoom Video Communications, Inc., San Jose, CA, USA). An interview guide (Supplemental Table 2) was developed based on the four objectives of the study and was designed to facilitate open-ended discussions while allowing for the emergence of new themes. Two female researchers (MKi, MKa) were present at interviews – one as the primary interviewer and the other as an observer. Both researchers are Georgian nationals with medical background (MD, MPH and MD, MSc, respectively), but neither had attempted nor enrolled in a medical residency program in Georgia and at the time of the study, both were employed at research institutions.

### Data Analysis

We chose reflexive thematic analysis, as outlined by Braun and Clarke,^7^ for its flexibility and systematic approach in identifying patterns and themes within qualitative data. Data analysis was conducted using NVivo 14 software (QSR International Pty Ltd, Melbourne, Australia) by two researchers (MKi, MKa) working in parallel, followed by consensus meetings to resolve any discrepancies. Data saturation was assessed after each interview, beginning with the fifth. Following ten interviews, both researchers agreed that no new information was emerging, and the decision was made to conclude data collection. Interview transcripts were retained in Georgian, the native language of both the participants and the researchers conducting the analysis. However, codes and themes were derived in English, as all authors are fluent in English. The average word count per interview transcript was approximately 2500 words. Findings are supported by illustrative quotes, translated from Georgian to English, to aid interpretation and enhance the credibility of the results. Additional visualizations representing the quantitative distribution of codes and themes are provided in Supplemental Figure 1.

## Results

Table 1 presents the demographic and background characteristics of the study participants. The mean age was 28 years, and 40% of participants were male. Three participants were not currently enrolled in a residency program, while the remaining seven were enrolled in either state (n = 5) or private (n = 2) programs. 50% of the participants had taken the residency placement exam more than once. All but one participant was based in Tbilisi, although several had relocated there for educational purposes – a plausible trend given that most medical training opportunities in Georgia are concentrated in the capital. Through thematic analysis, we identified six overarching themes: 1) quality of the Unified Postgraduate Qualification Examination; 2) navigating the enrollment process; 3) financial burden and employment before and during residency; 4) emotional stress; 5) perceptions about the residency programs; and 6) alternatives, including migration abroad or transitioning to another profession. Table 2 provides detailed description of these themes, along with the associated codes, the number of contributing participants, the number of transcripts linked to each code, and a representative illustrative quote. These themes are further elaborated in the text. Supplemental Figure 1 displays a hierarchy map that visually represents the volume of coded excerpts within each theme.

**Table 1.** Descriptive Characteristics of the Participants.

Pseudonym	Gender	Age	Currently Living and Working in	MD Degree Obtained in	Currently Enrolled in a Residency Program	Year(s) Taking the Placement Exam*	Year Enrolling in the Residency Program
Nino	Female	31	Tbilisi, Georgia	Private university	Yes, private	2020, 2021	2021
Mariam	Female	31	Tbilisi, Georgia**	State university	Yes, state	2017	2020
Giorgi	Male	25	Tbilisi, Georgia**	State university	No	2022, 2023	Not enrolled
Kristina	Female	32	Batumi, Georgia	Private university	No	2018, 2020, 2023	Not enrolled
Lika	Female	25	Tbilisi, Georgia	State university	No	2023	Not enrolled
Marika	Female	27	Tbilisi, Georgia	Private university	Yes, state	2022	2022
Ana	Female	30	Tbilisi, Georgia**	State university abroad	Yes, state	2019, 2020	2021
Levani	Male	27	Tbilisi, Georgia	State university	Yes, state	2022,2023	2023
Nika	Male	28	Tbilisi, Georgia	State university	Yes, state	2021	2021
Sandro	Male	24	Tbilisi, Georgia	State university	Yes, private	2023	2023

*Might be multiple if the participant retook the exam due to either not passing or with an aim to improve the score.

**Originally not from Tbilisi (moved for medical education and intends to stay for residency).

**Table 2.** Themes, Initial Codes, and Their Characteristics.

Themes	Codes	N of Participants (N = 10)	N of Transcript Excerpts (N = 134)	Sample Quote (Translated from Georgian)
Quality of the Unified Postgraduate Qualification Examination	The need to memorize the “open” questions	10	13	*“I just sat at the computer every day for an hour and memorized the open database. There is no other way to prepare for this exam”*
	Exam does not reflect knowledge	10	12	*“This exam does not demonstrate knowledge. If you took [a student] who just graduated and knows the textbooks, they would still not score high because it's not about the knowledge”*
	Wrong or outdated test questions	8	11	*“Some of the questions have explicitly wrong answers. We just all know that we must memorize the wrong answer”*
	Cheating and appeals	5	5	*“A person next to me was blatantly using a mobile phone. I personally know people who got very high scores due to cheating”*
	Role of private tutoring	3	3	*“Of course, I don’t have anything against people going to private groups to prepare, but no one knows what's going on in those groups, I know that some groups have access to stolen “closed” questions and sometimes even sell them”*
Navigating the enrollment process	Distribution of available positions	8	15	*“I wanted to study pulmonology, and only state programs have it. [However], they don’t announce positions every year, so I must wait”*
	Lack of transparency in private program selection process	6	9	*“You’re always in a waiting mode, trying to find out when [private programs] will announce positions. And even then, it might already be occupied”*
	State versus private residency programs: pros and cons	5	7	*“I aimed to get a high score to enroll in a state program, because the tuition fee is much lower than in private ones”*
Financial burden, Employment before and during residency	Financial burden	6	12	*“They’re telling us we must work, and we have to pay for this at the same time. This is a very unusual form of slavery”*
	Working during the residency, outside the residency program	6	8	*“Where I work, they teach me much more than at my residency program. I’d rather skip the program hours and work to have income and learn more”*
	Working before the residency	4	6	*“If you’re not a resident, it's very hard to get employment as a junior doctor. It's easier to be a nurse and it pays more too”*
Emotional stress	Helplessness and disappointment	4	4	*“I really want to be a doctor. It is really unfair and disappointing that we must go through all this”*
	Worrying and anxiety	4	4	*“I skipped the spring session because I was emotionally affected by the experience, this was too stressful”*
Other perceptions about the residency programs	Nepotism	7	7	*“Nepotism is everywhere in Georgia, as long as you have the right relatives in the right places.”*
	Poor quality of training and mentorship	3	5	*“I didn’t expect the quality of training to be good anywhere…* *it is unlikely to find a good mentor”*
Alternatives: moving abroad or to another profession	Alternative destinations or professions	4	6	*“To be honest, I’ve never thought of giving up the profession, I don’t see myself in any other job”*
	Ties to Georgia as a home country and community	3	3	*“I do not want to be without my friends and family abroad”*
	The US as a common destination	3	4	*“I’ve passed USMLE exams and am trying to match at a residency in the US”*

### Quality of the Unified Postgraduate Qualification Examination

All participants expressed skepticism about the *Unified Postgraduate Qualification Examination*, questioning both its contents and organization. The need to memorize the “open” question bank, which constitutes 75–80% of the exam, was widely viewed as non-educational: *“…it was so frustrating to just sit and memorize the answers, it gives you nothing.”* Several participants mentioned that memorizing test questions appeared easier for graduates of universities where reliance on “open” test banks was common practice, compared to graduates from other institutions. Some also noted that many questions were outdated or that some of the officially provided “correct” answers were, in fact, incorrect: “*Some questions have wrong answers, and you just need to memorize it in a wrong way.”*

Participants further emphasized that the exam did not adequately assess clinical knowledge or other relevant competencies, and that preparation for it using modern textbooks was of limited value. Concerns regarding cheating and corruption, particularly related to the “closed” portion of the exam, were also raised. Some reported witnessing cheating attempts during the exam, such as the use of mobile phones, while other alleged that portions of the “closed” question banks were available through private tutoring groups: “*I know that some tutoring groups have the closed database for sale…* *which is so unfair.”*

### Navigating the Enrollment Process

Respondents universally noted that the state-sponsored residency programs are limited in number and highly competitive, often requiring test scores in the high 190 s out of 200. A commonly discussed issue related to enrollment was the inconsistent distribution of available positions across specialties: *“the positions are available sporadically. For example, there might be 40 positions in a certain discipline one year, and 0 in the other.”* This lack of consistent availability led some participants to apply for alternative specialties with the hopes of transferring later to their preferred field: “*I’m studying sports medicine because there was no position announced in pulmonology, and my score was not high enough for internal medicine.”*

Participants expressed clear views regarding the choice between state- and privately sponsored residency programs. The advantages of state programs, as described by respondents, included lower tuition fees, fewer attendance requirements, the flexibility to work elsewhere during residency hours, and a transparent enrollment process based solely on exam scores: “*the enrollment process at the state spots is 100% transparent, it's online bidding, so you can see everyone's scores.”* However, the state-provided positions were perceived as extremely limited and highly competitive, particularly in some specialties: “*…for the state residency in internal medicine, they only post 2–3 spots a year, so there is no way of getting that unless you have a perfect score.”*

Respondents expressed several concerns about privately offered residency programs. One key issue was the absence of a centralized platform to monitor private residency vacancies and standardize the application process. These vacancies are currently announced through social media or on institutional websites, making it difficult to track opportunities consistently. Other commonly cited disadvantages included significantly higher tuition fees and rigid expectations on attendance: “*you’re basically working at the facility; you cannot do anything else.”* Additionally, participants unanimously agreed that the enrollment process at private facilities is far from transparent: “*they announce spots as they like, and internal exams are not available for review or appeals, I was not allowed to see my own test results”*.

### Financial Burden, Employment Before and During the Residency

More than half of the respondents highlighted financial concerns as the primary challenge associated with residency training.

Employment-related issues were relevant both before and after enrollment, as residency programs in Georgia do not provide salaries, and the tuition fees – particularly in some private facilities–can be prohibitively high. Respondents cited figures of 1000 GEL (about $360 per month in 2024). Participants widely viewed it as unfair that they are expected to pay for their training while receiving no compensation for their work: “*They’re telling us we must work, and we have to pay for this at the same time. This is a very unusual form of slavery.”*

Several respondents compared the Georgian context to international standards: “*it would be fair to be reimbursed for our work, as it happens in most developed countries like the US and Europe.”* Some noted that the combination of high tuition fees and strict attendance requirements in private facilities effectively excludes residency-seekers from lower socioeconomic backgrounds: “*I cannot pay the [private] fee and remain a burden to my family until I am in my thirties.”*

Participants who were unable to secure residency placements immediately after graduation highlighted the difficulties in finding employment: “*If you’re not a resident, it's very hard to get employment as a junior doctor. It's easier to be a nurse and it pays more too.”* In some cases, respondents revealed that they gained more practical knowledge and experience from their jobs than from their residency programs: “*where I work, they teach me much more than my residency program. I’d rather skip the program hours and work to have income and learn more.”* It was unanimously agreed that working during residency was more manageable for residents in state-sponsored programs, whereas private facilities imposed stricter attendance requirements and required residents to work at their institutions without pay. Some respondents reported taking jobs outside the medical field while continuing their efforts to secure a residency position.

### Emotional Stress

Several respondents spoke about the emotional impact of navigating the residency enrollment process. Some of the emotional experiences corresponded to the feelings of helplessness and disappointment with the system: “*I really want to be a doctor. It is really unfair and disappointing that we must go through all this.”* Others expressed feelings of worrying and anxiety: “*this was a period of extreme anxiety for me”.* One respondent expressed extremely disappointed after being unable to secure a job while waiting for residency positions – an outcome perceived to be influenced by nepotism in employment decisions.

### Other Perceptions About the Residency Programs

Opinions about the quality of residency programs were mixed. Some participants perceived private programs to be of higher quality, while others considered state-sponsored positions to be more “prestigious”. A common concern across both types of programs was the lack of structured mentorship. The quality of mentorship was described as either poor or dependent entirely on chance: “*it's really a lottery, depending on who you get as a mentor”.* One participant shared a sense of being an “extra” at their facility, since no one took an active interest in training them or assigning meaningful tasks. Another went so far as to characterize the entire process as akin to “*buying a license,”* since everything “*happens on paper”*. The issue of nepotism was raised by the majority of participants, citing instances where private residency positions were allegedly pre-assigned to the relatives of facility staff. These individuals were perceived to receive preferential treatment in terms of training opportunities. One respondent cited issue of nepotism as a contributing factor for poor-quality healthcare in the country: “*hospitals are full of incompetent relatives of influential doctors, who subsequently harm the patients.”*

### Alternatives: Moving Abroad or to Another Profession

More than half of the respondents had either considered or were actively taking steps toward emigrating to another country to pursue residency training. The United States was the most frequently mentioned destination, as several medical universities in Georgia offer English-language medical programs and incorporate preparation for the *United States Medical License Examination* (USMLE) in their curricula. Italy, Israel, and Belarus were mentioned as alternative destinations.

However, some respondents expressed a desire to remain in Georgia due to financial constraints or personal commitments: “*I’ve never considered USMLE exams, one for financial reasons, it's so expensive to take them, but also because I don’t want to leave my family and friends.”* While none of the participants had considered changing professions, some noted that if they were unable to secure a residency position “*while they’re still young,”* they might be forced to reevaluate their career paths.

## Discussion

In this interview study, we explored the experiences of ten residency-seekers and current medical residents in Georgia. Participants reported similar experiences regardless of gender, age, and residency status. All participants expressed critical views about the current system, mainly citing issues such as the poor quality of the placement exam, lack of coordination in the distribution of residency training opportunities across specialties, financial burdens due to unpaid residency positions, and the presence of nepotism and other unfair practices in medical training. Several participants also described the emotional toll the process had taken on them. More than half of the respondents reported considering emigration due to these challenges, raising concerns about a potential “brain drain” of medical graduates in Georgia. Based on the findings, we identified key areas for improvement and provide recommendations below, considering the specific context of the Georgian healthcare system.

Participants deplored the lack of high-quality training opportunities, despite Georgia having one of the highest physician densities in the world, peaking at 75.6 physicians per 10,000 people in 2018.^9^ This oversupply licensed physicians may have contributed to the oversaturation of clinical departments, thereby limiting residents’ opportunities to assume meaningful clinical responsibilities. The peculiar structure of medical specialization in Georgia further exacerbates the issue. Fields that are typically considered subspecialties of internal medicine in Europe and USA – such as cardiology, pulmonology, infectious diseases (ID), nephrology, endocrinology^10,11^) – are classified as standalone specialties in Georgia.^12^ As a result, a resident's practice is strictly limited to a single department. For example, an ID resident may only work within the ID department and provide ID-specific care, since residents are not permitted to deliver general internal medicine services, even when doing so may benefit the patient; only external consults may provide care outside a department's designated specialty. This non-holistic structure further limits the scope of residency training and contributes to the perception among residents that they are viewed essentially as “surplus” workforce. The oversaturation of clinical roles by licensed physicians may also contribute to the absence of financial compensation for residents, as little substantive work is available for them. Despite this, programs still expect residents to work while charging tuition fees. This long-standing status quo has likely fostered a culture of non-reimbursement. It is also important to note that almost all private residency facilities in Georgia operate as for-profit institutions, which may create additional incentives to use residents as unpaid labor to maximize institutional profitability.

One of the primary concerns raised by participants was the lack of salaries for residents, as residency in Georgia is not regarded as employment but rather as a training opportunity that requires the payment of tuition fees. Although there have been limited efforts to introduce targeted subsidies on tuition fees. In 2019, the MoH initiated funding for four designated priority residency programs: psychiatry, phthisiatry (standalone program for tuberculosis care), pediatric phthisiatry, and laboratory medicine. However, uptake of this initiative was extremely low, and the subsidized funding accounted for only 0.8% of total volume of tuition fees between 2018–2022.^4^ Furthermore, the mechanisms and criteria used to identify and designate these priority programs were not made transparent.

The distribution of training opportunities across specialties was heavily criticized by respondents. Due to limitations in data quality and accessibility, it is difficult to determine the exact mismatch between the number of medical graduates and available residency placements each year. However, the *2024 State Audit* report indicates that between 2018 and 2022, a total of 7051 students graduated from MD programs. While some of these graduates – particularly those enrolled in English-language programs – may not have intended to remain in Georgia, only 1595 were enrolled in residency programs during the same period, representing just 37% of total graduates.^4^ While the number of medical graduates may be disproportionately high, leading to a persistent shortage of residency spots, it remains crucial to develop a transparent and evidence-based strategy for determining specialty quotas. Such a strategy should be informed by the broader context of health service delivery in Georgia, including the hyper-specialization of medical professions, which contributes to elevated specialist fees, and ineffective gatekeeping at the primary care level by family physicians, due to high burden of work and prevailing perceptions about low-quality of primary care.^3,13^

**Figure 2.**
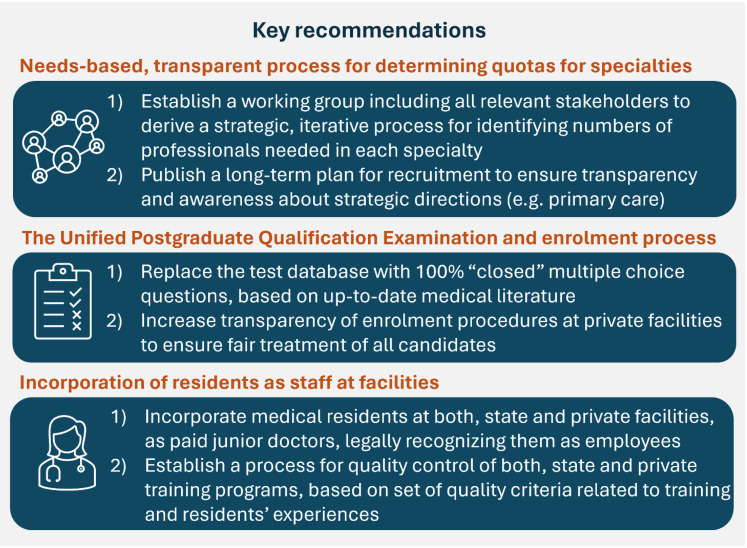
Key recommendations to the Ministry of Internally Displaced Persons from the Occupied Territories, Health, Labour, and Social Affairs of Georgia, based on the findings of our study as well as the recent State Audit report (February 2024).

Based on our findings and the recent State Audit Report,^4^ the MoH should develop a targeted strategy for medical residency reform, focusing on three key areas of improvement (Figure 2). First, the quotas for specialties – that is the number of residency positions available annually in both state and private facilities – should be determined through an iterative, strategic planning process involving all relevant stakeholders. This process should be coordinated by the MoH and include representatives from professional associations, private facilities offering residency programs, employers, and development partners providing technical assistance (eg, methods for assessing and predicting population health care needs). A long-term strategic plan should be developed and made publicly available to reduce confusion and disappointment among residency-seekers caused by the current lack of clarity around available training opportunities. Second, the examination and enrollment process must be reformed. Internationally, the post-graduate examination systems and practice requirements vary, reflecting differences in health workforce planning priorities and implementation.^14^ The challenges within Georgia's current system are well-documented, underscoring the need for the second key improvement area. The MoH should procure and maintain an up-to-date, validated, and confidential (not public) test database, that may either be developed domestically (as is done for the *Unified National Examinations* used for university admissions) or adapted from international sources with appropriate translation and contextualization. Additionally, the enrollment process at private facilities should be subject to regulation and transparency rules. Specifically, a centralized platform should be established where all private facilities are required to publicly announce available positions, outline their examination and interview procedures, and provide candidates with relevant preparatory materials. In addition, the selection process should be fair, consistent, and transparent. Facilities must also provide feedback to candidates regarding their performance and the basis for final decisions.

Finally, residents should be integrated into the healthcare system as staff members, with their labor legally recognized and appropriately compensated. They should be paid as “junior doctors” and receive the same employment benefits available to other healthcare workers. The majority of our respondents reported significant financial strain due to the burden of tuition fees and the necessity of working additional jobs to support their training. While implementing this change may present challenges–particularly given existing budgetary constraints and perceptions regarding residents’ “place” within the system–it is both unethical and inefficient to continue treating residents as “extra” staff with undefined roles and responsibilities, and no compensation.

The classification of residents as employees–with corresponding rights and remuneration–varies internationally and the topic remains underexplored in academic literature. In the United States, the status and rights of resident has been the subject of long-standing debate.^15–17^ Additionally, resident physician burnout, often stemming from poor working conditions and excessive working hours, has been shown to negatively impact the quality of care.^18,19^ At the same time, efforts to limit residents’ working hours have raised concerns about potential compromises in the quality of training.^20^ In the light of these policy changes, legal precedents, and resident right advocacy, there has been a shift towards recognizing residents as workers–an essential component of the health workforce–rather than merely learners or trainees, and extending them full employee rights.^17,21,22^

Difficulties with medical residency structures are prevalent in many post-Soviet states; however, up-to-date published research on this topic remains scarce. Descriptive studies from Armenia cite similar challenges, including the lack of strategic oversight in determining the number of resident physicians admitted per specialty and the lack of compensation during residency training.^23,24^ Some post-Soviet countries have preserved more elements of the Soviet model of medical education. For example, in Tajikistan, all MD graduates undertake a one-year clinical internship, which gives them a right to work as primary care physicians. Those wishing to specialize in secondary care must accumulate three years of experience in primary care and pass an oral, subjective examination.^25^ In contrast, the Baltic post-Soviet states have adopted more equitable and strategic approaches to organizing residency enrollment. In Latvia, the Ministry of Health annually determines the number of government subsidized (paid) residency positions for each specialty, based on hospital demand, population health needs, and the existing distribution in each specialty.^26^ In Estonia, all residents receive a monthly salary and are formally considered regular staff members.^27^

Several policy-level initiatives create a solid foundation for much needed reforms in Georgia's medical residency system. Notably, the *National Health Strategy of Georgia 2022–2030* urges for the enhanced collaboration with the *World Federation for Medical Education* (WFME), including the alignment of postgraduate education with WFME standards.^28^ The objective is to bring all residency programs in compliance with relevant WFME standards by 2030. The strategy also outlines tasks such as improving residency enrollment procedures, prioritizing specialties based on demand, and determining the appropriate number of training positions.^28^ To date, no interim assessments or operational plans have been made publicly available to evaluate progress towards these goals. The evidence presented in this study–highlighting the lived experienced of medical graduates in Georgia–can serve as a catalyst for driving policy reforms, particularly in a context where research resources remain limited.

### Strengths and Limitations

This data-driven study has several strengths. It is the first to explore the experiences of medical residency-seekers in Georgia, offering novel insights that could inform policy and decision-making in the country. The use of open-ended questions and a validated methodology for synthesizing findings using NVivo software enhances the reliability of the data. Additionally, our recruitment strategy was designed to ensure diversity within the sample, including participants both enrolled and not enrolled in residency programs, representing different genders and a range of medical universities.

Several limitations must be acknowledged. First, while analysis suggested that data saturation was reached, the overall sample size was limited. In particular, the number of participants enrolled in private residency programs was too small to comprehensively capture the variability across institutions in terms of enrollment procedures, tuition fees, and attendance requirements. Additionally, only one respondent was based outside of Tbilisi, which may limit the generalizability of the findings to other regions of Georgia. Further limitations include the lack of up-to-date national data on the mismatch between the number of medical graduates and available residency positions, as well as the absence of information on when specific residency examinations were last updated. Lastly, the high percentage of respondents considering emigration should be interpreted cautiously, as our sample may have been biased toward individuals who are internet-savvy, actively seeking information about residencies, and more aware of international opportunities.

## Conclusions

This study illustrated the significant challenges faced by residency-seekers and current medical residents in Georgia. Our findings reveal widespread dissatisfaction with the residency enrollment system, highlighting issues such as the poor quality of the placement exam, lack of coordination in the distribution of training opportunities, poor quality of training and lack of structured mentorship, financial hardship due to unpaid residencies, and the presence of nepotism and unfair practices. These challenges have not only imposed a substantial emotional burden on many residents but have also contributed to a concerning trend of potential “brain drain”, as many participants expressed intentions to seek training opportunities abroad due to their disillusionment with the current system.

Strategic reforms need to address transparent and needs-based long-term planning for residency quotas, a reformed examination and enrollment process, and improve the legal and financial integration of residents into the healthcare system. Aligning these reforms with future human resources for health planning efforts, the *National Health Strategy of Georgia 2022–2030,* and the standards of the *World Federation for Medical Education–*and fully implementing them–can drive significant improvements, ensuring that residency programs meet international standards and better serve the healthcare needs of the Georgian population.

## Supplemental Material

sj-docx-1-mde-10.1177_23821205251342050 - Supplemental material for Access to Medical Residency: A Qualitative Study of Medical Graduates' Experiences in Georgia Supplemental material, sj-docx-1-mde-10.1177_23821205251342050 for Access to Medical Residency: A Qualitative Study of Medical Graduates' Experiences in Georgia by Mariam Kirvalidze, Mariam Kasrashvili, Aleksandre Tskitishvili, Giorgi Aladashvili, Nikoloz Chelidze, Nikoloz Tvildiani, Karsten Lunze and Ilia Nadareishvili in Journal of Medical Education and Curricular Development

## References

[1] Curatio International Foundation. Human Resources in Healthcare: Situational Analysis [Internet]. Curatio International Foundation; 2018 [cited 2024 May 15]. (Health Barometer Studies). Available from: https://curatiofoundation.org/wp-content/uploads/2018/03/HRH_Barometer-10.pdf

[2] Minister of Labour, Health and Social Affairs of Georgia. Order of Minister of Georgia 295/ერთიანი დიპლომისშემდგომი საკვალიფიკაციო გამოცდის ჩატარების წესისა და პირობების და რეზიდენტურაში ჩარიცხვის წესის დამტკიცების შესახებ [On the Unified Postgraduate Qualification Examination Conducting Rules and Conditions and Rules of Enrollment in Residency] [Internet]. 2006. Available from: https://matsne.gov.ge/ka/document/view/67552

[3] Richardson N Berdzuli N. Georgia : Health System Review [Internet]. World Health Organization; 2017 [cited 2024 May 20]. (Health systems in Transition). Available from: https://who-sandbox.squiz.cloud/__data/assets/pdf_file/0008/374615/hit-georgia-eng.pdf 29972130

[4] State Audit Office of Georgia. Efficiency Report on Healthcare Workforce Development [Internet]. State Audit Office of Georgia; 2024 Feb [cited 2024 May 15]. Available from: https://shorturl.at/lowCI

[5] Euronews Georgia. Those who care about the medical residency. 2024 Mar 1 [cited 2024 Jul 11]; Available from: https://euronewsgeorgia.com/2024/03/01/sisac-rezidentura-adardebs/

[6] Radio Liberty Georgia. Junior Doctors: why do some people quit medical education in Georgia. 2023 Jun 3 [cited 2024 Jul 11]; Available from: https://www.radiotavisupleba.ge

[7] Braun V Clarke V . Using thematic analysis in psychology. Qual Res Psychol. 2006;3(2):77-101.

[8] Tong A Sainsbury P Craig J . Consolidated criteria for reporting qualitative research (COREQ): a 32-item checklist for interviews and focus groups. Int J Qual Health Care. 2007;19(6):349-357. 17872937 10.1093/intqhc/mzm042

[9] World Health Organization. WHO Dashboard. Density of physicians (per 10 000 population). [Internet]. World Health Organization Data. 2023 [cited 2024 May 20]. Available from: https://data.who.int/indicators/i/217795A.

[10] American College of Physicians. acponline.org. [cited 2024 Jun 4]. Subspecialties on Internal Medicine. Available from: https://www.acponline.org/about-acp/about-internal-medicine/subspecialties-of-internal-medicine

[11] Cranston M Slee-Valentijn M Davidson C Lindgren S Semple C Palsson R . Postgraduate education in internal medicine in Europe. Eur J Intern Med. 2013;24(7):633-638. 24028928 10.1016/j.ejim.2013.08.006

[12] Ministry of Internally Displaced Persons from the Occupied Territories, Labour, Health and Social Affairs of Georgia. Order of Minister of Georgia 136/საექიმო სპეციალობათა, მომიჯნავე საექიმო სპეციალობათა და სუბსპეციალობების შესაბამისი ნუსხის განსაზღვრის შესახებ [On the determination of medical specialties’, para-medical specialties’ and subspecialties’ list] [Internet]. Apr 18, 2007 p. 7. Available from: https://tsmu.edu/ts/images/file/1645104679brZaneba136_nTebervali2022.pdf

[13] World Health Organization. Georgia: moving from policy to actions to strengthen primary health care [Internet]. World Health Organization; 2023 [cited 2024 May 22]. (Primary heath care policy paper series). Available from: https://www.who.int/europe/publications/i/item/WHO-EURO-2023-7565-47332-69449

[14] Price T Lynn N Coombes L , et al. The international landscape of medical licensing examinations: a typology derived from a systematic review. Int J Health Policy Manag. 2018;7(9):782-790. 30316226 10.15171/ijhpm.2018.32 PMC6186476

[15] Geiger SL . The ailing labor rights of medical residents: curable ill or a lost cause? U Pa J Bus L. 2006;8(2):523-541.

[16] Mason MV. Are residents considered students or employees? JAMA. 1998;279(20):1668f.

[17] Care G . Are medical residents and fellows employees or students? Internet. 2024 [cited 2024 Jul 10]. Available from: https://browngold.com/blog/are-medical-residents-and-fellows-employees-or-students/.

[18] Barger LK Weaver MD Sullivan JP Qadri S Landrigan CP Czeisler CA . Impact of work schedules of senior resident physicians on patient and resident physician safety: nationwide, prospective cohort study. bmjmed. 2023;2(1):e000320. 10.1136/bmjmed-2022-000320 PMC10254593 37303489

[19] Ludmerer KM . Resident burnout: working hours or working conditions? J Grad Med Educ. 2009;1(2):169-171. 21975971 10.4300/JGME-D-09-00077.1 PMC2931255

[20] Nagasaki K Kobayashi H . The effects of resident work hours on well-being, performance, and education: a review from a Japanese perspective. J of Gen and Family Med. 2023 Nov;24(6):323-331. 10.1002/jgf2.649 PMC10646297 38025934

[21] Hilker S Butler A. Valuing the Resident Physician: Advocating for Change. NEJM Resident 360 [Internet]. 2023 May 10 [cited 2024 Jul 10]; Available from: https://resident360.nejm.org/expert-consult/valuing-the-resident-physician-advocating-for-change

[22] Fujikawa H Son D Eto M . Are residents learners or workers? A historical perspective in Japan. TAPS. 2021;6(1):122-124.

[23] Markosian C Sargsyan G Shariff S , et al. Medical education in Armenia: an overview. J Med Educ Curric Dev. 2023;10(10):23821205231203831. 37868044 10.1177/23821205231203831 PMC10588409

[24] Markosian C Shekherdimian S Badalian SS Libaridian L Jilozian A Baghdassarian A . Medical education in the former soviet union: opportunities in Armenia. Ann Glob Health. 2020;86(1):99. 32864351 10.5334/aogh.2960 PMC7427658

[25] Zavadskiy SP Yefimova AM . Medical education state reform in Tajikistan: between tradition and modernity. Med Teach. 2020;42(8):861-870. 32476521 10.1080/0142159X.2020.1767284

[26] Jain N Jersovs K Safina T , et al. Medical education in Latvia: an overview of current practices and systems. Front Med. 2023;10(10):1250138. 10.3389/fmed.2023.1250138 PMC10551541 37809335

[27] European Junior Doctors. European Junior Doctors Network - Estonia [Internet]. [cited 2024 May 23]. Available from: https://www.juniordoctors.eu/medicalmobility/estonia#:∼:text=No%20fee%20for%20residency%20programmes.&text=Residents%20are%20paid%20monthly%20salaries,money%20or%20additional%20free%20time.

[28] Ministry of Internally Displaced Persons from the Occupied Territories, Labour, Health and Social Affairs of Georgia. ჯანმრთელობის. დაცვის ეროვნული:სტრატეგია. Available from: https://www.moh.gov.ge/uploads/files/2022/gancxadebebi/NHS_short.pdf. [National Health Strategy] 2022-2030 [Internet]. 2022.

