# Supplementary material for: Access to Medical Residency: A Qualitative Study of Medical Graduates' Experiences in Georgia
Source: J Med Educ Curric Dev. 2025 May 11;12:23821205251342050. doi: 10.1177/23821205251342050 (PMC12066863; doi:10.1177/23821205251342050)

**Supplementary Table 1**. Consolidated Criteria for Reporting Qualitative Studies (COREQ): 31-item checklist.

| **Item** | **Question** | **Page** |
| --- | --- | --- |
| **Domain 1: Research team and reflexivity** | | |
| 1. Interviewer/facilitator | Which author/s conducted the interview or focus group? | 3 |
| 2. Credentials | What were the researcher’s credentials? E.g. PhD, MD | 3 |
| 3. Occupation | What was their occupation at the time of the study? | 3 |
| 4. Gender | Was the researcher male or female? | 3 |
| 5. Experience and training | What experience or training did the researcher have? | 3 |
| 6. Relationship established | Was a relationship established prior to study commencement? | NA |
| 7. Participant knowledge of the interviewer | What did the participants know about the researcher? e.g. personal goals, reasons for doing the research | NA |
| 8. Interviewer characteristics | What characteristics were reported about the interviewer/facilitator? e.g. Bias, assumptions, reasons and interests in the research topic | NA |
| **Domain 2: Study design** | | |
| 9. Methodological orientation/Theory | What methodological orientation was stated to underpin the study? e.g. grounded theory, discourse analysis, ethnography, phenomenology, content analysis | 3 |
| 10. Sampling | How were participants selected? e.g. purposive, convenience, consecutive, snowball | 3 |
| 11. Method of approach | How were participants approached? e.g. face-to-face, telephone, mail, email | 3 |
| 12. Sample size | How many participants were in the study? | 3 |
| 13. Non-participation | How many people refused to participate or dropped out? Reasons? | NA |
| 14. Setting of data collection | Where was the data collected? e.g. home, clinic, workplace | 3 |
| 15. Presence of non-participants | Was anyone else present besides the participants and researchers? | NA |
| 16. Description of sample | What are the important characteristics of the sample? e.g. demographic data, date | 4 |
| 17. Interview guide | Were questions, prompts, guides provided by the authors? Was it pilot tested? | 3 |
| 18. Repeat interviews | Were repeat interviews carried out? If yes, how many? | NA |
| 19. Audio/visual recording | Did the research use audio or visual recording to collect the data? | 4 |
| 20. Field notes | Were field notes made during and/or after the interview or focus group? | NA |
| 21. Duration | What was the duration of the interviews or focus group? | 3 |
| 22. Data saturation | Was data saturation discussed? | 4 |
| 23. Transcripts returned | Were transcripts returned to participants for comment and/or correction? | NA |
| **Domain 3: Analysis and findings** | | |
| 24. Number of data coders | How many data coders coded the data? | 4 |
| 25. Description of the coding tree | Did authors provide a description of the coding tree? | 4 |
| 26. Derivation of themes | Were themes identified in advance or derived from the data? | 4 |
| 27. Software | What software, if applicable, was used to manage the data? | 4 |
| 28. Participant checking | Did participants provide feedback on the findings? | NA |
| 29. Quotations presented | Were participant quotations presented to illustrate the themes / findings? | 4 |
| 30. Data and findings consistent | Was there consistency between the data presented and the findings? | 4 |
| 31. Clarity of major themes | Were major themes clearly presented in the findings? | 4,5,6 |
| 32. Clarity of minor themes | Is there a description of diverse cases or discussion of minor themes? | 4,5,6 |

**Supplementary Table 2**. Interview Guide (translated from Georgian).

| **Study objectives** | **Guiding question**  Keep it open-ended, avoid giving options but provide examples to clarify, if necessary |
| --- | --- |
| 1) Explore the experiences of medical graduates in navigating the residency enrollment process | How did you experience the entire residency enrollment process? (e.g., exam, application period, interviews, matching, feedback) |
| 2) Understand the emotional, psychological, and practical aspects involved in preparing for and participating in residency placement examinations and enrollment | How did it feel to go through the entire residency enrollment process? (e.g., excitement, anxiety, worry, other emotions) |
| 3) Identify areas of improvement within the residency enrollment process | What would you like to be done differently in the process? (e.g., what would help make it accessible and equitable) |
| 4) Explore attitudes towards the opportunities to enroll in residency programs abroad (i.e., “brain drain”). | Have you ever considered moving abroad for medical residency? Where and why? |

**Supplementary Figure 1.** A hierarchy map demonstrating the volume of excerpts corresponding to the codes within each theme.
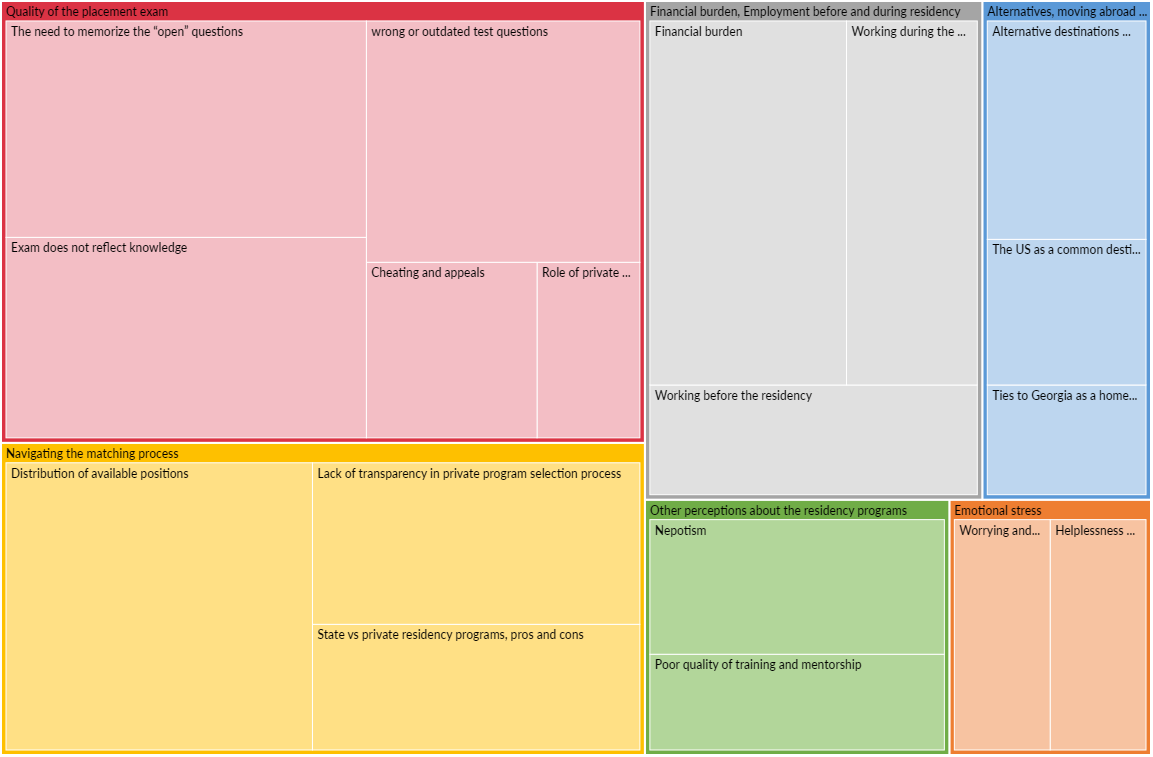

Supplement: sj-docx-1-mde-10.1177_23821205251342050 - Supplemental material for Access to Medical Residency: A Qualitative Study of Medical Graduates' Experiences in Georgia [file sj-docx-1-mde-10.1177_23821205251342050.docx]
